# Retraction: Deletion of the scavenger receptor Scarb1 in osteoblast progenitors does not affect bone mass

**DOI:** 10.1371/journal.pone.0290458

**Published:** 2023-08-16

**Authors:** Michela Palmieri, Teenamol E. Joseph, Charles A. O’Brien, Horacio Gomez-Acevedo, Stavros C. Manolagas, Elena Ambrogini

Following the publication of this article [1], an error was identified in regard to the selection of the mouse model.

Specifically, the authors had sought to delete Scarb1 in cells of the osteoblast lineage but used the C57BL/6N-Scrib<tm1c(NCOM)Mfgc>/Tcp conditional allele which encodes the protein Scrib, not the protein Scarb1.

The authors would also like to alert the readership that the same error occurred in another of their recent articles, which has also been retracted [2]. A reader of the article in *Bone* [2] identified the error.

The authors regret this error that nullifies the conclusions of the paper. In light of this issue, the authors retract the article.

All authors agree with the retraction and apologize for the issues with the published article.
